# Addressing COVID-19 Misinformation on Social Media Preemptively and Responsively 

**DOI:** 10.3201/eid2702.203139

**Published:** 2021-02

**Authors:** Emily K. Vraga, Leticia Bode

**Affiliations:** University of Minnesota, Minneapolis, Minnesota, USA (E.K. Vraga);; Georgetown University, Washington, DC, USA (L. Bode)

**Keywords:** social media, coronavirus, communication, World Health Organization, infections, COVID-19, viruses, severe acute respiratory syndrome coronavirus 2, SARS-CoV-2, coronavirus disease

## Abstract

Efforts to address misinformation on social media have special urgency with the emergence of coronavirus disease (COVID-19). In one effort, the World Health Organization (WHO) designed and publicized shareable infographics to debunk coronavirus myths. We used an experiment to test the efficacy of these infographics, depending on placement and source. We found that exposure to a corrective graphic on social media reduced misperceptions about the science of 1 false COVID-19 prevention strategy but did not affect misperceptions about prevention of COVID-19. Lowered misperceptions about the science persisted >1 week later. These effects were consistent when the graphic was shared by the World Health Organization or by an anonymous Facebook user and when the graphics were shared preemptively or in response to misinformation. Health organizations can and should create and promote shareable graphics to improve public knowledge.

The uncertainty around the emergence of severe acute respiratory syndrome coronavirus 2, a novel coronavirus that causes coronavirus disease (COVID-19), has led to the rapid and widespread diffusion of misinformation about the virus, its origins, and effective prevention and treatment strategies (*1*,*2*). Misinformation is not a new problem, but it poses particular challenges for infectious disease management when public acceptance is required for prevention behaviors such as social distancing or wearing a mask.

As part of the effort to promote good information over misinformation, the World Health Organization (WHO) has created and publicized shareable infographics (“mythbusters”) that debunk specific myths about COVID-19 (*3*). Research regarding the efficacy of health organization websites designed to debunk misinformation has yielded mixed results. Material from the Centers for Disease Control and Prevention (CDC) regarding the influenza vaccine successfully reduced misperceptions that the vaccine can cause influenza or is unsafe but also reduced intentions to get the vaccine among those concerned about its side effects (*4*). Likewise, WHO material debunking Zika virus rumors did not affect most targeted misperceptions and also reduced the accuracy of related beliefs about Zika virus (*5*). These examples reinforce concern that repeating false information, even to correct it, can strengthen belief in the myths (*6*,*7*).

In this study, we considered the effectiveness of sharing WHO’s myth correction graphics on social media specifically. This project differed from previous research in 2 ways. First, the graphic used in every correction was clearly labeled as coming from WHO, which may boost effectiveness compared with research that did not prominently display the source of the corrective material (*4*,*5*). Second, we considered exposure to someone sharing a specific correction graphic on social media, rather than to website material more generally. Previous research has found that observational correction, which occurs when persons see misinformation being corrected on social media and update their own attitudes in response, is effective for emerging infectious disease topics such as Zika virus (*8*,*9*) and for infectious diseases such as influenza (*10*). We aimed to determine the effectiveness of social media sharing of a graphic that debunks 2 related coronavirus myths.

## Methods

### Study Design

In this study we considered the effectiveness of sharing a WHO graphic (on social media) that debunks 2 related coronavirus myths: that taking a hot bath both raises body temperature and prevents coronavirus infection (Figure). Scientific evidence suggests that hot baths can minimally affect body temperature; studies have found a change of roughly 0.5°C –1.0°C in body temperature (*11*,*12*). Temperatures needed to deactivate coronavirus are typically >56°C (*13**–**15*), which exceed safe bath temperatures; scalding is likely within 10 minutes at 48°C (*16*). In other words, this graphic explains the science for why hot baths do not prevent COVID-19 and directly disputes the prevention efficacy of baths. The graphic follows many best practices for combating misinformation: it is fact-based, colorful, simple, and easy to understand; focuses on the fact rather than the myth; and includes a label signaling that it comes from an expert source (*7*,*9*,*10*). These aspects fulfill many of the 5 Cs of correction: is consensus based, includes corroborating evidence, and is consistent, coherent, and credible (*6*). Addressing the science behind why hot baths do not prevent COVID-19 infection also corroborates the argument with a science-based alternative explanation shown to boost correction effectiveness (*6*,*7*,*17*). Therefore, we expected that exposure to a post containing this graphic would reduce the 2 misperceptions among persons targeted by the graphic as compared with persons who did not see any information on the topic.

**Figure:**
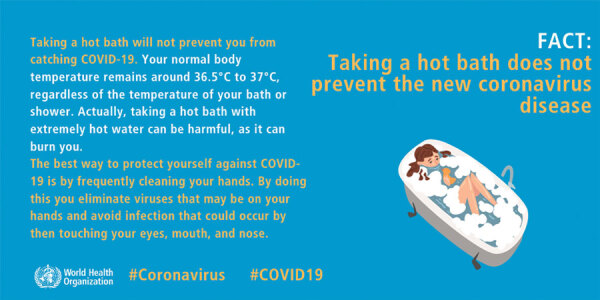
Original World Health Organization myth buster graphic used in study of addressing COVID-19 misinformation on social media. COVID-19, coronavirus disease.

Such a graphic might be shared in multiple ways, which we also tested. The first factor manipulates whether the graphic was shared preemptively on a social media feed, compared with whether it was shared in response to misinformation on the topic (we refer to this as placement). When offered preemptively, a user shares the graphic as a social media post without addressing the misinformation directly. In this case, it might function like a fact check, addressing an inaccurate claim made elsewhere but not directly linking to that claim on the social media platform (*18**–**20*). Alternatively, the graphic could be shared in response to someone posting misinformation. These responsive corrections are a relatively common behavior (*21*) and reduce belief in misinformation among other social media users who witness the correction (*8*,*9*,*22*). Given the relative dearth of research in this space, we explored whether preemptive or responsive posting strategies are more effective in reducing misperceptions.

The second factor manipulates who shares the information. Previous research on correction has emphasized the ability of an expert source like WHO to address misinformation (*7*,*22*,*23*) but offers mixed evidence about the effectiveness of a single user in correcting misinformation on social media (*22*,*24*). Therefore, we expect that a graphic shared by WHO will more effectively reduce misperceptions than the same graphic (still with WHO branding) shared by an unknown Facebook user.

In addition, we explored the combination of these 2 elements: who shared a graphic and whether it was shared in response or preemptively. Although it is not clear how these 2 elements interact, several possibilities seem plausible. For instance, it might seem strange to see a powerful organization like WHO responding directly to misinformation, making this form of correction less effective for WHO but not for users. Alternatively, research suggests that a user debunking a myth preemptively using facts might be less effective than when sharing a correction after misinformation (*24*), but we do not have research to determine whether this pattern should similarly hold for organizations. Although research does not clearly specify what to expect, the interaction between source and type of sharing is worth exploring.

Finally, not enough correction research has been done to investigated the enduring effect of exposure to misinformation and its correction. Some research suggests that corrections fade over time, and the myth could actually be reinforced through an illusory truth effect of seeing misinformation repeated (*6*,*7*). Alternatively, if the correction follows best practices by emphasizing facts and providing an alternative explanation, as we believe the WHO graphic does, lowered misperceptions may endure over time. Therefore, we tested whether the effects of correction endure over 1 week.

### Experimental Design

An experimental design enabled us to best consider the effects of who corrected and whether the correction was in response to misinformation or independent of it. This experiment received approval from the Institutional Review Board at the University of Minnesota on April 27, 2020.

We fielded a survey experiment to 1,596 participants during May 4–5, 2020 (wave 1) using Amazon’s Mechanical Turk service (https://www.mturk.com). Of these, 1,453 were willing to continue participation and 1,419 passed an attention check in the first wave of the study; these participants were contacted 1 week later (on May 12, with a recontact on May 14) for a follow-up survey (wave 2). A total of 1,122 participants (79%) completed wave 2 an average of 7.5 days later (mean 7.54, SD 0.75).

Each participant viewed a screenshot of a Facebook feed and was asked to read it as if it were on their own feed (Appendix 1). The experiment consisted of 6 experimental conditions (Appendix 2): a pure control condition, a misinformation-only condition, and 4 correction conditions manipulated in a crossed factorial design with the 2 factors we described earlier: placement (preemptive versus responsive) and source (WHO versus user).

In the pure control condition, participants viewed 5 control posts on the simulated feed. In the misinformation-only condition, they viewed the same 5 posts, with the addition of a misinformation post: a status posted by a user saying “This is such an easy thing to do! Take a hot bath to keep yourself healthy and protect you from coronavirus!” on a bright pink background.

For all correction conditions, participants viewed the same WHO infographic, which prominently labels the source, to isolate the effects of who is sharing the graphic rather than the graphic itself. Those who viewed the preemptive correction saw the correction infographic as the second post in the feed, posted either by WHO or by a social media user but with no misinformation post as part of the feed. Those who viewed the responsive correction saw the misinformation post described earlier, with the corrective graphic posted in response, either by a user or by WHO in the form of a WHO “info bot.” Although no such bot exists as far as we know, WHO and Facebook have partnered to offer a Facebook messenger bot to answer user questions about coronavirus (*25*), so this sort of correction is plausible, if not currently being deployed. Moreover, a bot offers a scalable and realistic responsive mechanism, rather than assuming that WHO would directly respond to individual Facebook users on their official feeds.

After exposure to the simulated Facebook feed in wave 1, participants answered questions regarding their beliefs regarding the myths targeted by the WHO graphic to measure misperceptions about body temperature and COVID-19 prevention (Appendix 3). These questions were replicated in wave 2 of the study.

### Sample Characteristics

Of the 1,596 participants who completed our initial survey, participants skewed male (62.9%) and highly educated (72% had a bachelor’s degree or higher). Participants averaged 37 years of age (mean 36.94 years, SD 11.31 years), were relatively diverse in terms of race and ethnicity (18.5% African-American, 7.9% Asian-American, 70.6% White; 21.3% considered themselves Hispanic or Latino) and income (median $50,000–$75,000) and leaned Democratic (5-point scale, mean 3.73, SD 2.00) and liberal (5-point scale, mean 3.69, SD 1.93). These characteristics were consistent among participants who completed the second wave of the study (Appendix 2 Table 1).

### Statistical Analysis

We performed 2 sets of analyses based on our preregistration (*26*). First, we compared each of the experimental conditions to the pure control condition using linear regression to determine whether the corrections reduced misperceptions as compared with baseline beliefs (absent any information regarding hot baths or COVID-19). We replicated these analyses for wave 2. Second, we isolated the effects of source and placement using a regression approach (not preregistered) excluding both the control and misinformation-only conditions, and entering 2 factors (placement and source) as well as the interaction between the two.

## Results

### Wave 1

First, we tested the effects of correction on misperceptions related to the effects of a hot bath on body temperature and COVID-19 prevention for wave 1. We limited these regression analyses to the 1,543 persons who passed a premanipulation attention check (Appendix 4). Exposure to the WHO graphic in any condition reduced misperceptions that a hot bath will raise body temperature as compared with the control, but had no effects on misperceptions that a hot bath will prevent COVID-19 infection (Table 1). When comparing the types of correction to each other, we found no differences by either source or placement, nor by the interaction between the 2 categories (Table 2). In other words, corrections were equally effective for body temperature misperceptions (and ineffective for COVID-19 prevention misperceptions) whether they came from a user or from WHO and when they were preemptive as well as responsive.

**Table 1:** Comparing participants in correction conditions to control condition for wave 1 using regression analysis in study of addressing COVID-19 misinformation on social media*

Condition	**Body temperature**			**COVID-19 prevention**	
	Beta	SE		Beta	SE
Pure control [reference]	–	–		–	–
Misinformation only	–0.06	0.08		–0.13	0.09
WHO preemptive	–0.40‡	0.09		–0.12	0.09
User preemptive	–0.26†	0.08		–0.10	0.09
WHO responsive	–0.46‡	0.08		–0.14	0.09
User responsive	–0.30‡	0.08		–0.05	0.09
Adjusted R^2^	0.028‡			0.000	

*Adjusted R^2^ indicates the variance explained by the overall model. COVID-19, coronavirus disease; WHO, World Health Organization.  **†**p<0.01. ‡p<0.001.

**Table 2:** Comparing participants among the 4 correction conditions for wave 1 using regression analysis in study of addressing COVID-19 misinformation on social media*

Condition	Body temperature			COVID-19 prevention	
	Beta	SE		Beta	SE
WHO (vs. user)	–0.13	0.11		–0.03	0.11
Responsive (vs. preemptive)	–0.04	0.11		0.05	0.11
Interaction	–0.03	0.15		–0.06	0.16
Adjusted R^2^	0.002			0.000	

*Adjusted R^2^ indicates the variance explained by the overall model. COVID-19, coronavirus disease; WHO, World Health Organization.

### Wave 2

We replicated these analyses with the 1,110 participants who completed the follow-up survey and passed the attention check for wave 2 (12 participants failed the attention check in wave 2), controlling for the amount of time between taking the 2 waves of the survey. We found that exposure to the WHO preemptive, WHO responsive, or user responsive corrections all produced lower misperceptions than the control condition at wave 2 for body temperature misperceptions (Table 3). We also found that those exposed to the WHO responsive correction had significantly lower COVID-19 prevention misperceptions 1 week later than those in the control condition; results showed an average decline of 11% in COVID-19 prevention misperceptions from the control to the WHO responsive correction. However, the overall model predicting COVID-19 misperceptions was not significant, meaning that there were no differences in means averaged across the 6 experimental conditions even though there was a significant difference in directly comparing the WHO responsive correction to control condition, so this result must be interpreted with caution. We again found no significant differences in either type of misperceptions based on the source of the graphic (WHO versus Facebook user) or whether it was offered preemptively or responsively (Table 4).

**Table 3:** Comparing participants in correction conditions to control condition for wave 2 using regression analysis in study of addressing COVID-19 misinformation on social media*

Condition	Body temperature			COVID-19 prevention	
	Beta	SE		Beta	SE
Time gap	0.05	0.04		–0.01	0.04
Pure control [reference]	–	–		–	–
Misinformation only	–0.09	0.10		–0.20	0.10
WHO preemptive	–0.29†	0.11		–0.13	0.11
User preemptive	–0.08	0.11		–0.09	0.10
WHO responsive	–0.35†	0.11		–0.22‡	0.10
User responsive	–0.21‡	0.11		–0.10	0.11
Adjusted R^2^	0.010†			0.001	

*Adjusted R^2^ indicates the variance explained by the overall model. COVID-19, coronavirus disease; WHO, World Health Organization. **†**p<0.01. **‡**p<0.05.

**Table 4:** Comparing participants among the four correction conditions for wave 2 using regression analysis in study of addressing COVID-19 misinformation on social media*

Condition	**Body temperature**			**COVID-19 prevention**	
	Beta	SE		Beta	SE
Gap	0.09	0.06		–0.01	0.06
WHO (vs. user)	–0.21	0.13		–0.05	0.13
Responsive (vs. preemptive)	–0.14	0.13		–0.01	0.13
Interaction	0.08	0.19		–0.07	0.18
Adjusted R^2^	0.006			0.000	

*Adjusted R^2^ indicates the variance explained by the overall model. COVID-19, coronavirus disease; WHO, World Health Organization.

## Discussion

Efforts to address misinformation on social media have taken on special urgency with the emergence of COVID-19. Mitigating the risks associated with COVID-19 requires sustained public action, so misinformation that promotes false preventives or cures can hinder necessary behaviors to reduce the spread of the disease. In this study, we tested whether sharing graphics from WHO designed to address COVID-19 misinformation can reduce misperceptions. Our results suggest that although these graphics do not affect all misperceptions, reductions in misperceptions that do occur persist over time.

Notably, exposure to the WHO graphic in any form reduced immediate misperceptions about the science of a false preventive for COVID-19 (that a hot bath can raise body temperature), and this reduction was maintained for at least 1 week for 3 of the 4 correction conditions. This finding suggests that understanding of the science behind why hot baths do not prevent COVID-19 prevention does not deteriorate rapidly.

Although these effects on reducing science-related misperceptions show the promise of the WHO graphics as myth busters on social media, we did not see a parallel reduction in the related misperceptions regarding prevention efficacy (that a hot bath will prevent COVID-19 infection). We offer several post hoc explanations for these findings. First, we suspect that a floor effect may partially explain these null effects; even in the control condition in wave 1, participants were largely well informed, rating the argument that a hot bath can prevent COVID-19 infection as at least probably false (55.8% had an average score <2 or less on a scale of 1, definitely false, to 5, definitely true). In contrast, only 17.5% believed that the claim that a hot bath can raise body temperature was probably false, offering more leverage to change beliefs. Second, motivated reasoning may make persons more resistant to updating beliefs as issues around COVID-19 and the WHO become more politicized in the United States (*27*); this motivated reasoning is likely less operant for the science of why such prevention is not effective. Third, persons may have thought that the science regarding hot baths and their effects on body temperature is better established given longstanding research (*11*,*12*), boosting confidence in the validity of the correction. Given high levels of scientific as well as public uncertainty regarding COVID-19 (*28*), the public may have been less convinced regarding the scientific evidence that a hot bath does not prevent COVID-19.

Finally, the fact that a hot bath does not raise body temperature may not be the only (or even the most prominent) reason that persons may believe that taking a hot bath decreases the risk of COVID-19 infection. A supplemental analysis (Appendix 5 Table 1) provides some evidence for this explanation. In the pure control condition, the correlation between misperceptions that a hot bath raises body temperature and a hot bath can prevent COVID-19 is not significant (Pearson’s correlation coefficient *r =* 0.06; p = 0.16). In the misinformation-only condition, the correlation is not significantly stronger than in the control condition (p = 0.27). However, for both WHO correction conditions, the correlation is significantly stronger than both the pure control and misinformation conditions (p<0.05). This preliminary evidence suggests that the correction, especially when shared by WHO, helps participants mentally link the science claim and the prevention claim; however, this explanation accounts for, at most, 18% of variance in COVID-19 prevention beliefs. Therefore, the explanation for why hot baths do not prevent COVID-19 is not the only factor in persons’ beliefs about prevention efficacy.

These effects were consistent whether the graphic was shared by WHO itself or by another user. We suspect the similar effects between users and WHO, in contrast to earlier research suggesting experts were more effective than users (*22*,*23*), may result from the prominent labeling of WHO within the graphic itself, boosting the credibility of the post. Therefore, mobilizing users to share WHO’s graphics may produce similar effects in reducing misperceptions.

We found limited evidence that preemptive corrections differ in their effectiveness from reactive corrections. Preemptive and responsive corrections are equally effective when considering whether hot baths affect body temperature, both immediately and over time. Likewise, both are unsuccessful in affecting misperceptions about the efficacy of hot baths to prevent COVID-19 infection immediately after exposure to the correction. If preemptive corrections are effective in reducing misperceptions for (some) myths, persons need not wait until seeing someone share misinformation but can share the posts created by official expert organizations to address misperceptions in society at large. Thus, more attention is needed to find ways to motivate persons to share these types of corrections on their feeds.

However, the reactive correction addresses both the prevention efficacy of a hot bath (which is raised by the misinformation post) and the science behind this explanation, which is not addressed in the misinformation post. If the misinformation had also offered an explanation for why a hot bath supposedly reduces COVID-19 risk through raising body temperature, perhaps a reactive correction would be more effective. Although research suggests that false cures and preventives are a major subset of COVID-19 misinformation (*2*), these studies do not elaborate on whether the misinformation contains false claims about the science behind the myth. We suspect that providing false explanations is a subset of misinformation claims and therefore chose to have the misinformation post include only the COVID-19 prevention myth to enhance external validity. Best practices for correction suggest that including an alternative explanation and corroborating evidence enhances the power of corrections (*6*,*7*,*17*). Furthermore, emerging research suggests that correcting a related myth not raised in the misinformation can reduce misperceptions on that related myth, serving as an alternative form of preemptive correction (*29*).

We did find 1 case in which a responsive correction from WHO may be more effective than the other corrections: exposure to the WHO responsive condition reduces misperceptions that a hot bath can prevent COVID-19 infection as compared with the control condition 1 week later, although this result must be interpreted with caution given the insignificance of the model overall and the limited amount of variance explained. If this result holds, it could be that the WHO responsive condition is the most memorable, and therefore had the most lasting effect on misperceptions, which future research should test.

We also found that both body temperature and COVID-19 prevention misperceptions were lower in wave 2 than in wave 1 for both the control and misinformation conditions (Appendix 5 Table 2). We suspect that the debriefing that all participants viewed at the end of wave 1 of the study, which included the WHO graphic and explained the myth, functioned as a correction itself (as intended to reduce potential misperceptions). Therefore, it is noteworthy that some correction conditions reduced hot bath misperceptions even further in wave 2 compared with the control, which reinforces the value of multiple corrections (*7*,*22*).

This study’s limitations suggest caution in interpreting our findings. First, we relied on a diverse but unrepresentative sample of the US public, most notably skewing educated and male. Future research should explore these effects among a representative sample and samples outside the United States, including countries where the worst of the pandemic has passed and ones that are struggling to contain new outbreaks, to examine how these contexts affect the relationships we observed here. Second, although our study suggests that the WHO graphics have potential given their effects on body temperature misperceptions, low levels of initial belief that hot baths can prevent COVID-19 limited our ability to perceive potential effects on prevention efficacy. Similarly, the post promoting misinformation about hot baths preventing COVID-19 was largely not persuasive in generating misperceptions. Future research should consider efforts to debunk more prominent or plausible COVID-19 myths. Third, we selected a myth with little partisan divide; we cannot speak to whether these graphics would be effective for politically polarized myths (*11*). Fourth, the effect sizes explained were relatively small, so corrections should be deployed as part of a larger health communication strategy for promoting accurate COVID-19 information.

Despite these limitations, this study offers several practical and theoretical advancements. First, we found little evidence of a backfire effect in promoting misperceptions of sharing the WHO’s infographics on social media. This finding not only fits with increasing evidence about the rarity of backfire effects (*30*) but is also reassuring that sharing the graphics at least does no harm. Second, we find that preemptively sharing these graphics can be effective. Users and organizations can debunk misinformation circulating in society by sharing high-quality information on social media emphasizing the facts without waiting to see it shared directly in their feeds, which expands the opportunities for observational correction to occur. Third, we found that a WHO bot that directly responds to misinformation may be a particularly effective technique. Partnerships with platforms may enable these automated responses to prominent myths, furthering the reach of expert organizations. Creating easily shared graphics that promote facts in spaces in which misinformation abounds appears promising as part of a broader strategy to enable more efficient and effective corrections on social media.

Appendix 1 Graphics showing experimental stimuli for the study of COVID-19 misinformation on social media.

Appendix 2 Sample characteristics across waves in the study of COVID-19 misinformation on social media.

Appendix 3 COVID-19 prevention misconceptions given in the study of COVID-19 misinformation on social media.

Appendix 4 Attention checks in the study of COVID-19 misinformation on social media.

Appendix 5 Additional analyses for the study of COVID-19 misinformation on social media.
