# Supplementary material for: Addressing COVID-19 Misinformation on Social Media Preemptively and Responsively
Source: Emerg Infect Dis. 2021 Feb;27(2):396–403. doi: 10.3201/eid2702.203139 (PMC7853571; doi:10.3201/eid2702.203139)
Supplement: Appendix 1 — Graphics showing experimental stimuli for the study of COVID-19 misinformation on social media. [file 20-3139-Techapp-s1.pdf]

Article DOI: https://doi.org/10.3201/eid2702.203139

Addressing COVID-19 Misinformation on Social Media Preemptively and Responsively

Appendix 1

Experimental Stimuli

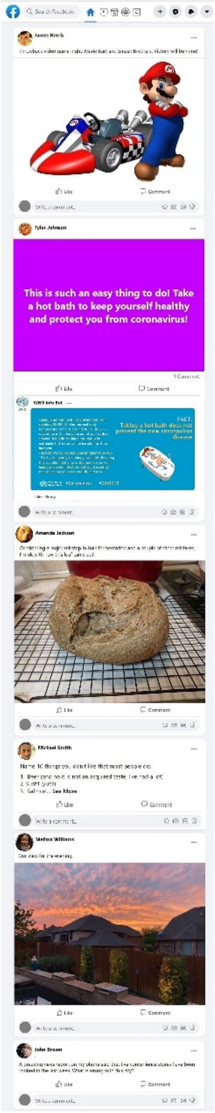

Appendix 1 Figure 1. Experimental stimuli from WHO responsive condition, showing full feed.

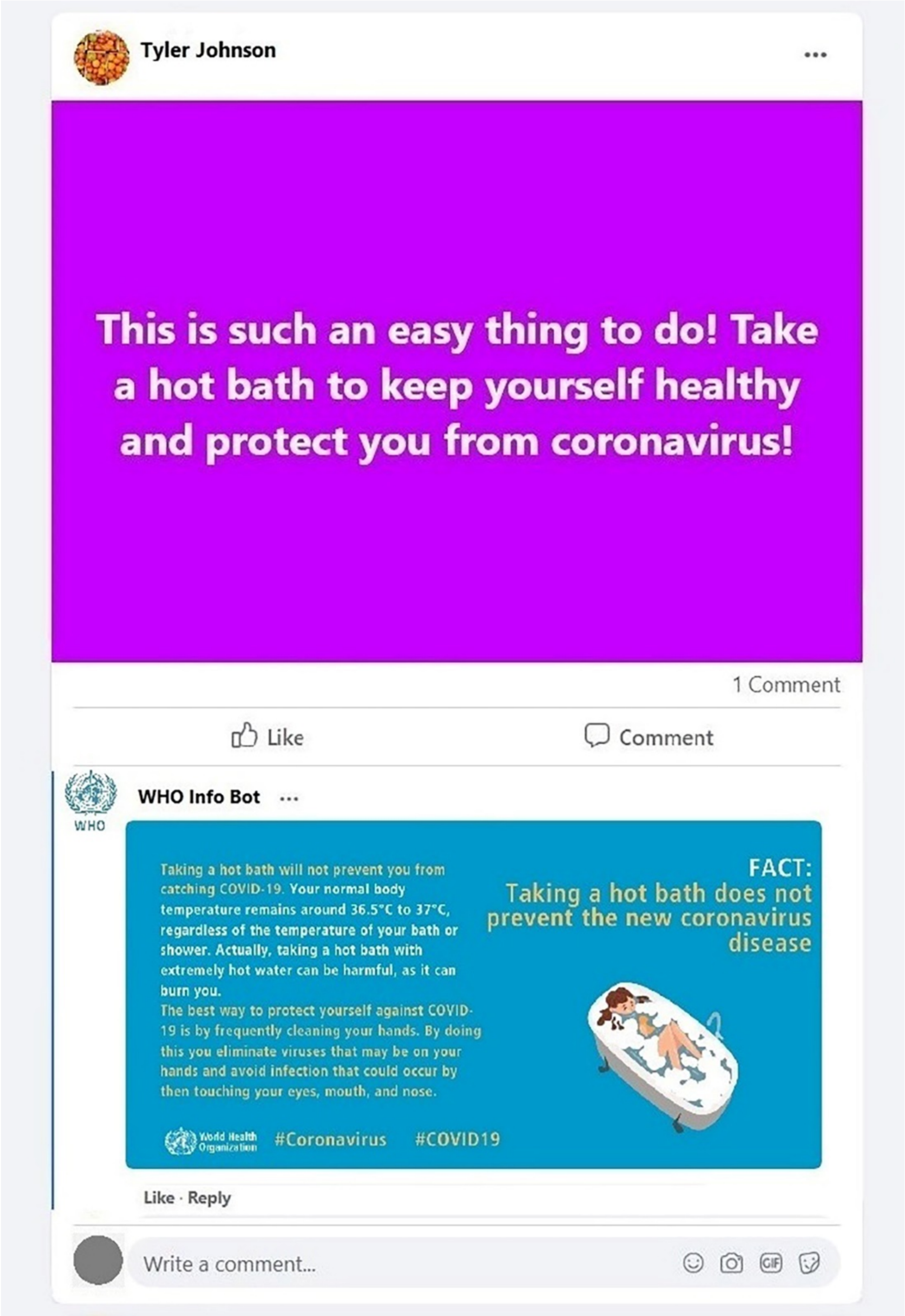

Appendix 1 Figure 2. Experimental stimuli from WHO responsive condition, showing only misinformation and correction.
