# Supplementary material for: Addressing COVID-19 Misinformation on Social Media Preemptively and Responsively
Source: Emerg Infect Dis. 2021 Feb;27(2):396–403. doi: 10.3201/eid2702.203139 (PMC7853571; doi:10.3201/eid2702.203139)
Supplement: Appendix 2 — Sample characteristics across waves in the study of COVID-19 misinformation on social media. [file 20-3139-Techapp-s2.pdf]

Article DOI: https://doi.org/10.3201/eid2702.203139

Addressing COVID-19 Misinformation on Social Media Preemptively and Responsively

Appendix 2

Sample Characteristics across Waves

Appendix 2 Table 1. Characteristics of sample participants in wave 1 and wave 2 of the study of COVID-19 misinformation in social media

	Wave 1		Wave 2	
Characteristic	**Mean**	**SD**	**Mean**	**SD**
Age	36.89	11.30	37.35	11.37
Education	4.62	1.16	4.59	1.18
Income	2.95	1.23	2.98	1.24
Party affiliation	3.73	1.99	3.73	1.99
Political ideology	3.69	1.93	3.71	1.91
Percentages				
Male	62.5		61.6	
Female	37.5		38.4	
White	71.7		72.3	
African-American	17.9		16.7	
Asian-American	7.8		8.7	
Hispanic or Latino	20.1		16.8	

Appendix 2 Table 2. Number of persons per experimental condition

Category	Wave 1	Wave 2
Control	599	432
Misinformation	200	144
WHO preemptive	181	130
User preemptive	187	134
WHO responsive	193	138
User responsive	183	132
