# Supplementary material for: Addressing COVID-19 Misinformation on Social Media Preemptively and Responsively
Source: Emerg Infect Dis. 2021 Feb;27(2):396–403. doi: 10.3201/eid2702.203139 (PMC7853571; doi:10.3201/eid2702.203139)
Supplement: Appendix 3 — COVID-19 prevention misconceptions given in the study of COVID-19 misinformation on social media. [file 20-3139-Techapp-s3.pdf]

Article DOI: https://doi.org/10.3201/eid2702.203139

Addressing COVID-19 Misinformation on Social Media Preemptively and Responsively

Appendix 3

Measures

COVID-19 Prevention Misperceptions

Measured on a 5-point scale, from definitely false to definitely true.

Wave 1: Cronbach's alpha = 0.85, mean = 2.07, SD = 1.07, n = 1,542

Wave 2: Cronbach's alpha = 0.87, mean = 1.85, SD = 1.06, n = 1,110

1. Taking a hot bath will prevent infection from COVID-19.
2. If someone is exposed to COVID-19, a hot bath may prevent illness.
3. Taking a hot bath has no effect on COVID-19 (reversed).
4. A hot bath kills the COVID-19 virus before it can infect you.

Body Temperature Misperceptions

Measured on a 5-point scale, from definitely false to definitely true.

Wave 1: Pearson's correlation $r = 0.35$, $p<0.001$, mean = 2.94, SD = 1.01, n = 1,542

Wave 2: Pearson's correlation $r = 0.37$, $p<0.001$, mean = 2.81, SD = 1.08, n = 1,110

1. Human body temperature is largely unaffected by bath temperature (reversed).
2. You can raise your body temperature by taking a hot bath.
