# Supplementary material for: Addressing COVID-19 Misinformation on Social Media Preemptively and Responsively
Source: Emerg Infect Dis. 2021 Feb;27(2):396–403. doi: 10.3201/eid2702.203139 (PMC7853571; doi:10.3201/eid2702.203139)
Supplement: Appendix 4 — Attention checks in the study of COVID-19 misinformation on social media. [file 20-3139-Techapp-s4.pdf]

Article DOI: https://doi.org/10.3201/eid2702.203139

Addressing COVID-19 Misinformation on Social Media Preemptively and Responsively

Appendix 4

Attention Checks

In wave 1 of the study, participants answered a question before the manipulation that asked, “How much of a threat, if any, do each of the following pose to **you personally**?” for a matrix of options, including COVID-19 (the disease caused by SARS-CoV-2), the economic downturn, government overreach, climate change, and terrorism. Response options were *none at all*, *a little*, *a moderate amount*, *a lot*, and *a great deal.* During this battery, an item asked participants to select “a little” for that question if they were paying attention. This item was randomized with the other options. Participants who did not select “a little” for this response (n = 53) were excluded from all analyses and were not invited to participate in wave 2 of the study.

In wave 2, participants answered a question before the manipulation that asked, “Overall, how would you rate the performance of each of the following groups in responding to COVID-19?” for a matrix of options, including the World Health Organization, the Centers for Disease Control and Prevention, the US federal government, their state government, and their local government. Response options were *terrible, poor, average, good,* and *excellent.* During this battery, an item asked participants to select “good” for that question if they were paying attention. This item was randomized with the other options. Participants who did not select “good” for this response (n = 12) were excluded from all analyses for wave 2.
