# Supplementary material for: Addressing COVID-19 Misinformation on Social Media Preemptively and Responsively
Source: Emerg Infect Dis. 2021 Feb;27(2):396–403. doi: 10.3201/eid2702.203139 (PMC7853571; doi:10.3201/eid2702.203139)
Supplement: Appendix 5 — Additional analyses for the study of COVID-19 misinformation on social media. [file 20-3139-Techapp-s5.pdf]

Article DOI: https://doi.org/10.3201/eid2702.203139

Addressing COVID-19 Misinformation on Social Media Preemptively and Responsively

Appendix 5

Additional Analysis

Appendix 5 Table 1. Correlations between science and prevention efficacy myths†

Characteristic	Wave 1
Control	0.06
Misinformation	0.15*
WHO preemptive	***0.37****
User preemptive	0.06
WHO responsive	***0.39****
User responsive	**0.24****

*p<0.05, **p<0.01, ***p<0.001
†Bolded values indicate correlation significantly stronger than pure control condition, p<0.05 using a Fisher r-to-z transformation, 2-tailed test. Italicized values indicate correlation is significantly stronger than misinformation condition, p<0.05, using Fisher r-to-z transformation, 2-tailed test

Appendix 5 Table 2. Comparing the means by experimental condition between wave 1 and wave 2*

Characteristic	Wave 1	Wave 2	Statistical test
Body temperature misperceptions			
Control	**3.12**	**2.94**	***t* = 4.15, p<0.001**
Misinformation	**3.08**	**2.85**	***t* = 2.98, p<0.01**
WHO preemptive	*2.71*	*2.64*	***t*** = 0.81, p = 0.42
User preemptive	2.91	2.86	***t*** = 0.60, p = 0.55
WHO responsive	*2.62*	*2.58*	***t*** = –0.50, p = 0.52
User responsive	*2.79*	*2.72*	***t*** = 0.86, p = 0.39
COVID-19 prevention misperceptions			
Control	**2.02**	**1.94**	***t* = 2.79, p = 0.01**
Misinformation	**1.89**	**1.74**	***t* = 2.75, p = 0.01**
WHO preemptive	1.86	1.80	*t* = 1.41, p = 0.16
User preemptive	1.95	1.85	*t* = 1.87, p = 0.06
WHO responsive	**1.85**	***1.72***	***t* = 2.69, p = 0.01**
User responsive	**2.01**	**1.83**	***t* = 3.31, p<0.01**

*Analysis limited to persons who passed both wave 1 and wave 2 attention checks to run a paired-samples *t*-test as the statistical test. Both COVID-19 misperceptions and body temperature misperceptions were measured on a 5-point scale from “definitely false” to “definitely true”; exact measures are found in Appendix 1. Bold values indicate a significant decline from wave 1 to wave 2 in misperceptions; italicized values represent a significantly different value from the control condition within that wave.
